# Impact of psychosocial stressors on type 2 diabetes among migrants and non-migrants in The Netherlands: The HELIUS study

**DOI:** 10.1016/j.jmh.2025.100330

**Published:** 2025-03-31

**Authors:** Daniela Del Carlo Gonçalves, Charles Agyemang, Eva L. van der Linden, Charles Hayfron Benjamin, Anja Lok, Henrike Galenkamp, Eric Moll van Charante, Felix P. Chilunga

**Affiliations:** a Department of Public and Occupational Health, Amsterdam Public Health Institute, Amsterdam UMC, University of Amsterdam, The Netherlands; b Department of Physiology, University of Ghana Medical School, Accra, Ghana; c Department of Psychiatry, Amsterdam Public Health Institute, Amsterdam UMC, University of Amsterdam, The Netherlands; d Department of General Practice, Amsterdam Public Health Institute, Amsterdam UMC, University of Amsterdam, The Netherlands

**Keywords:** Migration, Psychosocial stress, Type 2 diabetes

## Abstract

**Background:**

Migrant populations in Europe have a type 2 diabetes (T2D) burden two to five times that of non-migrants. However, the role of psychosocial stressors—whose experiences can uniquely vary across population groups—remains underexplored. We examined associations between work stress, home stress, and adverse life events with T2D across major ethnic groups in The Netherlands.

**Methods:**

We used baseline data from HELIUS cohort (2011–2015), including 21,501 adults of Dutch, Moroccan, Turkish, South-Asian Surinamese, African Surinamese, and Ghanaian origin. Psychosocial stress was assessed using validated measures in preceding 12 months. T2D was defined by World Health Organization criteria. Robust Poisson regression estimated prevalence ratios (PRs), adjusting for age, sex, and education. Mediation and moderation analyses explored behavioural pathways and role of social support.

**Results:**

Occasional work stress was inversely associated with T2D in total population (aPR 0.82; 95 % CI 0.75–0.93) and among Moroccan-origin participants [0.76 (0.63–0.97)]. Regular home stress was positively associated with T2D in total population [1.15 (1.03–1.28)], but not across ethnic groups. Adverse life events were linked to higher T2D risk overall [1.22 (1.03–1.41)], and among Dutch [1.48 (1.21–1.76)] and African Surinamese [1.43 (1.09–1.89)] origin populations. BMI and alcohol use partially mediated these associations. Social support buffered work and home stress.

**Conclusion:**

Work stress, home stress, and adverse life events differentially influence T2D risk in diverse populations, with effects pronounced in Dutch, Moroccan and African Surinamese origin groups. Interventions targeting psychosocial stress may help reduce T2D in diverse populations.

## Introduction

Migrant populations in Europe experience a disproportionately high burden of type 2 diabetes (T2D), with a two- to five-fold increased risk compared to non-migrant (majority) populations (Meeks et al., 2016). While established risk factors—such as genetics, body composition, unhealthy diet, smoking, alcohol use, and depression—have been implicated, they only partially explain this elevated risk (van der Kooi et al., 2015; van Leijden et al., 2018; van Etten et al., 2020; Muilwijk et al., 2022; Zethof et al., 2021; Huisman et al., 2018). This underscores the need to explore additional contributors. One such emerging factor is psychosocial stress, a non-traditional yet potentially significant determinant of T2D (Hackett and Steptoe, 2017).

Psychosocial stress has been linked to T2D through both direct and indirect mechanisms (Hackett and Steptoe, 2017; Kelly and Ismail, 2015). Directly, stress activates allostatic pathways, including stimulation of the central nervous system (CNS) and the hypothalamic-pituitary-adrenal (HPA) axis, leading to chronic low-grade inflammation, dysregulated glucose metabolism, neuroendocrine disturbances, and abnormal cortisol secretion patterns (Hackett and Steptoe, 2017; Kelly and Ismail, 2015). Indirectly, stress may promote adverse health behaviours—such as unhealthy eating, physical inactivity, smoking, and excessive alcohol use—that are well-established risk factors for T2D (Hackett and Steptoe, 2017; Kelly and Ismail, 2015).

Migrants in Europe may face unique and chronic stressors, often coupled with reduced social support, which may influence T2D risk differently than in the non-migrant population (Bustamante et al., 2017). These stressors may include financial strain from low-paying jobs (Kentikelenis et al., 2015), language barriers (Evenden et al., 2022), cultural dissonance (Bhugra, 2005; Borrell et al., 2015), and perceived discrimination (Snoubar and Zengin, 2022). Furthermore, recent migrants may have smaller social networks in their new environments, exacerbating the psychological burden of stress (Snoubar and Zengin, 2022).

To date, the link between psychosocial stress and T2D has primarily been studied in European majority populations, while research among migrant groups remains limited (Hackett and Steptoe, 2017; Kelly and Ismail, 2015). In these majority populations, home-related stress and adverse life events have consistently been associated with an increased risk of T2D (Kelly and Ismail, 2015). In contrast, findings on work-related stress have been inconsistent—some studies report a positive association (Nyberg et al., 2014), while others find no link (Sui et al., 2016). These inconsistencies have been attributed to differences in stress measurement, cultural interpretations, and variations in labour policies across settings (Nyberg et al., 2014; Sui et al., 2016).

In The Netherlands, major migrant populations—including individuals of Turkish, Moroccan, South Asian Surinamese, African Surinamese, and Ghanaian origin—bear a disproportionately high burden of T2D and face unique stressors, such as financial obligations to families abroad (Schans, 2009), employment in low-paying jobs due to lower educational attainment (De Lange et al., 2019), bereavement due to deaths abroad they cannot attend (Nesteruk, 2018), and perceived discrimination (Te Lindert et al., 2022). This presents a critical opportunity to investigate the role of psychosocial stressors in shaping ethnic disparities in T2D prevalence.

In our previous research, we found that perceived discrimination—one form of psychosocial stress—was associated with metabolic syndrome among migrant groups but not in the Dutch majority population (Schmengler et al., 2017). However, an assessment of other forms of psychosocial stress (e.g., stress at work, stress at home, and adverse life events) in relation to T2D is still lacking. Therefore, in the current study, we examined the associations between work-related stress, home-related stress, and adverse life events with T2D among migrant (Moroccan, Turkish, South Asian Surinamese, African Surinamese) and non-migrant (Dutch) populations in The Netherlands. We hypothesize that the associations between psychosocial stressors and T2D vary by migration background, based on the differing experiences and interpretations of stressors across migrant populations. In addition, we explored the mediation and moderation impact of lifestyle factors and moderation role of social support.

## Methodology

### Study design and population

This study is part of the Healthy Life in an Urban Setting (HELIUS) study, a multi-ethnic prospective cohort based in Amsterdam, The Netherlands, focusing on cardiovascular diseases, mental health outcomes, and infectious diseases (Stronks et al., 2013; Snijder et al., 2017). Data were collected at two time points: baseline (2011–2015) and follow-up (2019–2021). For the present analysis, only baseline data were used, as psychosocial stress factors were not assessed during the follow-up period (Stronks et al., 2013; Snijder et al., 2017). This is because the follow-up coincided with the COVID-19 pandemic, during which stress levels increased by at least 60 % and became intense and widespread across the population (Chilunga et al., 2022). As such, these levels were deemed unrepresentative of the everyday stressors that the questionnaires were originally designed to capture under normal life circumstances.

Detailed description of the HELIUS study has been provided elsewhere (Stronks et al., 2013; Snijder et al., 2017). In brief, the HELIUS study included a total of 24,789 persons at baseline, including people with a Dutch background along with individuals from the largest migrant groups in Amsterdam (African Surinamese, South-Asian Surinamese, Turkish, Moroccan, and Ghanaian origin populations) aged 18 to 70 years old. The participants were randomly sampled, stratified by ethnic group, from the Amsterdam municipal register. Migration background was defined according to the individual's country of birth and parents. Participants were considered first-generation immigrants if they were born abroad and had at least one parent born abroad, or second-generation if they were born in The Netherlands but both parents were born abroad. The migrants were predominantly first generation. All participants completed a self-administered questionnaire and underwent a physical examination during which biological samples were obtained. All measurements were done accordingly to standardised protocols (Stronks et al., 2013; Snijder et al., 2017).

### Ethical approval

The HELIUS study was approved by the Ethical Review Board of the Amsterdam University Medical Centers, location AMC. All participants gave informed consent prior to enrolment in the study.

### Psychosocial stressors

Psychosocial stress resulting from situations experienced at home or at work in the preceding 12 months was measured using the well-validated psychological stress scale created by the INTERHEART study (Appendix 1) (Rosengren et al., 2004). The psychological stress scale is well-recognized and widely used in the literature (Chilunga et al., 2023; Chilunga et al., 2019). Participants were asked separately about the presence of stress at home or work and could answer: ‘never’ = 1, ‘some periods’ = 2, ‘several periods’ = 3, or ‘constantly’ = 4. For stress at work, there was an additional category of ‘does not apply.’ This category was grouped with the ‘never’ category, as these participants did not also experience any work-related stress (Chilunga et al., 2023; Chilunga et al., 2019).

Adverse life events refer to significant negative experiences or challenges that individuals may encounter throughout their lives, including loss of a loved one, serious illness or injury, financial difficulties, relationship problems, and major life transitions. Adverse life events were measured through the well validated list of threatening experiences (LTE) (Appendix 2) (Brugha et al., 1985). Participants were asked about nine acute stress situations in the last 12 months and could answer ‘no’ = 0 or ‘yes’ = 1. For the analysis, participants were categorized as having experienced any or no adverse life events in the last 12 months, a widely used approach in the literature (Chilunga et al., 2023; Chilunga et al., 2019).

### Definition of type 2 diabetes

T2D was defined according to World Health Organization criteria: fasting plasma glucose ≥ 7.0 mmol/L (126 mg/dL), use of glucose-lowering medication, or self-reported physician diagnosis (Harris et al., 2000). Fasting blood samples were analysed spectrophotometrically using hexokinase-catalysed reactions. Participants were asked to bring all prescribed medications, which were verified and classified using the Anatomical Therapeutic Chemical (ATC) system.

### Covariates

Additional participant characteristics included age (in years), sex (male or female), and educational level, based on the highest qualification obtained in The Netherlands or country of origin, categorized as: (1) no schooling/elementary only, (2) lower vocational/lower secondary, (3) intermediate vocational or intermediate/higher secondary, and (4) higher vocational or university. Occupational level was classified using the Dutch Standard Occupational Classification system and grouped into elementary, lower, medium, higher, and scientific levels. Physical activity was assessed using the Short Questionnaire to Assess Health-enhancing Physical Activity (SQUASH) and categorized based on international guidelines (≥30 min of moderate-to-high intensity activity on ≥5 days per week) (Nicolaou et al., 2016). Smoking status was categorized as current, former, or never smoker, and alcohol use in the past 12 months as yes or no. Daily fruit intake was recorded as the average number of fruits consumed per day. Body mass index (BMI) was calculated as weight in kilograms divided by height in meters squared (kg/m²). Perceived and desired social support was measured using the Social Support Questionnaire Transactions (SSQT) and Satisfaction (SSQS) scales, and categorized as low, medium, or high based on the approach by Muilwijk et al. within the HELIUS cohort (Muilwijk et al., 2022).

### Statistical analyses

Data were analysed using RStudio (version 4.4.1). Normally distributed variables were summarized as mean ± standard deviation, skewed variables as median (IQR), and categorical variables as frequency (%). The outcome of interest was T2D status (present vs. not present), and the exposures were three psychosocial stressors: stress at work, stress at home, and adverse life events. Robust Poisson regression was used to estimate prevalence ratios (PRs), which provide accurate and interpretable estimates in cross-sectional analyses and account for heteroscedasticity.

Stressors were modelled as categorical variables. For stress at work and at home, the "constant" and "several periods" categories were combined due to the small number of participants reporting constant stress, which limited meaningful comparisons. Ultimately, we used three categories for both work- and home-related stress: none, some periods/occasional, and regular (a combination of constant and several periods). Analyses were first conducted in the total sample, followed by stratification by migration status after testing for interaction effects. All models were adjusted for age, sex, and education level. Occupational level was intentionally excluded from the models to avoid overadjustment of work-related stress effects. To assess the independent contributions of each stressor, all three were included simultaneously in the final model. Lifestyle factors—smoking, alcohol use, physical activity, fruit intake, and body mass index (BMI)—were not included in the main models, as they may act as mediators in the relationship between stress and T2D. Instead, their potential mediating roles were explored in a sensitivity analysis using the mediation package in R.

Based on previous findings from the HELIUS cohort showing that desired emotional support is inversely associated with T2D, we also tested interaction (moderation) effects between each psychosocial stressor and emotional support in relation to T2D. All results were reported as prevalence ratios (PRs) with 95 % confidence intervals (CIs). Model diagnostics included assessment of the dispersion ratios, deviance residuals, and Cook's distances to evaluate model fit and identify influential observations. All statistical tests were two-tailed with a significance level of α = 0.05.

## Results

### Baseline characteristics

**Fig. 1:**
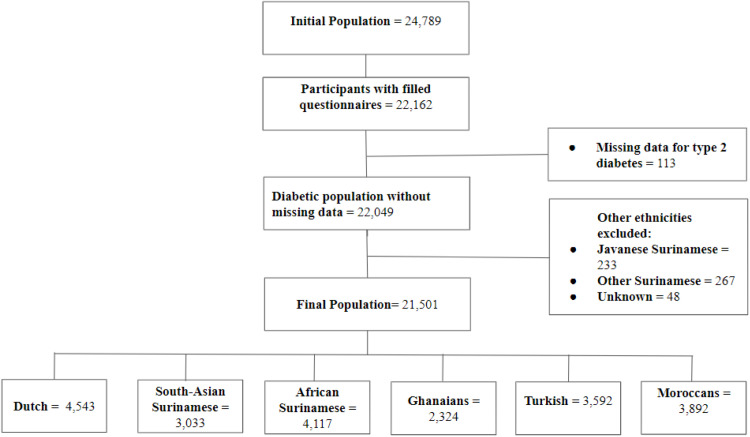
**Flowchart of participant inclusion.** This figure illustrates the selection process of participants, including inclusion and exclusion criteria, leading to the final analytic sample of 21,501 individuals from the HELIUS cohort.

Of the 24,789 HELIUS participants, 22,162 completed both the questionnaire and physical examination. We excluded 113 individuals with missing T2D data and an additional 548 due to small sample sizes and/or mixed or unspecified migration backgrounds (233 Javanese Surinamese, 267 other Surinamese, 48 other/unknown origin). The final analytic sample comprised 21,501 participants (Fig. 1): 4543 (21 %) Dutch, 3033 (14 %) South-Asian Surinamese, 4117 (19 %) African Surinamese, 2324 (11 %) Ghanaian, 3592 (17 %) Turkish, and 3892 (18 %) Moroccan origin.

Most participants were women (58 %), and the mean age was 44 years (SD = 13). Dutch origin participants had the highest proportions of higher education (61 %), high-level occupations (21 %), recommended physical activity (76 %), ex-smokers (38 %), alcohol use (91 %), and desired social support (63 %) compared to all other groups. Turkish participants had the highest mean body mass index (BMI), at 29 kg/m² (SD = 6), while the lowest was observed among Dutch origin participants (25 kg/m², SD = 4). Fruit intake of at least once per day was high across all ethnic groups, with >90 % meeting this threshold (Table 1).

**Table 1:** Baseline characteristics of participants.

Variables	Total *N* = 21,501	Dutch origin *N* = 4543	South-Asian Surinamese origin *N* = 3033	African Surinamese origin *N* = 4117	Ghanaian origin *N* = 2324	Turkish origin *N* = 3592	Moroccan origin *N* = 3892
**Sex, n ( %)**							
Male	908 1 (42.24)	2081 (45.81)	1366 (45.04)	1602 (38.91)	901 (38.77)	1623 (45.18)	1508 (38.75)
Female	12,420 (57.76)	2462 (54.19)	1667 (54.96)	2515 (61.09)	1423 (61.23)	1969 (54.82)	2384 (61.25)
**Age, mean (SD)**							
	44.26 (13.20)	46.19 (14.03)	45.52 (13.97)	47.92 (12.53)	44.76 (11.16)	40.36 (12.17)	40.46 (12.92)
**Generation of migration, n (%)**							
First generation	12,172 (77.67)	N/A	2322 (76.56)	3438 (83.51)	2218 (95.44)	2524 (70.27)	2670 (68.60)
Second Generation	3786 (22.33)	N/A	711 (23.44)	679 (16.49)	106 (4.56)	1068 (29.73)	1222 (31.40)
**Educational level, n (%)**							
Never been to school/Elementary	3803 (17.85)	150 (3.32)	436 (14.45)	229 (5.61)	657 (28.79)	1128 (31.74)	1203 (31.21)
Lower secondary	5595 (26.26)	643 (14.23)	1005 (33.31)	1462 (35.82)	912 (39.96)	881 (24.79)	692 (17.75)
Higher secondary	6194 (29.07)	990 (21.91)	881 (29.20)	1452 (35.58)	571 (25.02)	1015 (28.56)	1285 (33.33)
Higher vocational/University	5715 (26.82)	2735 (60.54)	695 (23.04)	938 (22.98)	142 (6.22)	530 (14.91)	675 (17.51)
**Occupational Level, n (%)**							
Elementary	1914 (16.07)	77 (1.80)	289 (10.82)	264 (7.08)	1247 (63.82)	535 (19.82)	502 (17.86)
Lower	5437 (29.98)	650 (15.23)	932 (34.91)	1317 (35.31)	457 (23.39)	1104 (40.90)	977 (34.76)
Medium	4791 (26.42)	999 (23.40)	832 (31.16)	1319 (35.36)	173 (8.85)	659 (24.42)	809 (28.78)
Higher	3636 (20.05)	1650 (38.65)	479 (17.94)	723 (19.38)	57 (2.92)	291 (10.78)	436 (15.51)
Scientific	1355 (7.47)	893 (20.92)	138 (5.17)	107 (2.87)	20 (1.02)	110 (4.08)	87 (3.09)
**Physical Activity, (n (%)**							
Not recommended level	9355 (43.57)	1110 (24.45)	1410 (46.58)	1590 (38.66)	1089 (46.88)	2088 (58.24)	2068 (53.23)
Recommended level	12,117 (56.43)	3429 (75.55)	1617 (53.42)	2523 (61.34)	1234 (53.12)	1497 (41.76)	1817 (46.77)
**Smoking, n (%)**							
Yes	5131 (23.97)	1119 (24.68)	857 (28.36)	1296 (31.64)	104 (4.51)	1235 (34.62)	520 (13.41)
No	12,002 (56.08)	1683 (37.12)	1752 (57.97)	2001 (48.85)	2012 (87.21)	1688 (47.32)	2866 (73.92)
Ex-smoker	4270 (19.95)	1732 (38.20)	413 (13.67)	799 (19.51)	191 (8.28)	644 (18.05)	491 (12.66)
**Alcohol consumption, n (%)**							
Yes	10,828 (50.64)	4130 (91.03)	1701 (56.31)	2805 (68.65)	1095 (47.61)	811 (22.76)	286 (7.38)
No	10,554 (49.36)	407 (8.97)	1320 (43.69)	1281 (31.35)	1205 (52.39)	2752 (77.24)	3589 (92.62)
**BMI (kg/m^2^), mean (SD)**							
	27.11 (5.28)	24.76 (4.19)	26.32 (4.83)	27.81 (5.53)	28.48 (4.98)	28.55 (5.72)	27.57 (5.21)
**Fruit intake (per day), n (%)**							
2 or more pieces of fruit a day	11,092 (53.44)	2002 (44.93)	1598 (54.67)	2414 (61.18)	1229 (56.30)	1960 (56.39)	1889 (50.09)
1 piece of fruit a day	8449 (40.71)	2296 (51.53)	1163 (39.79)	1338 (33.91)	809 (37.06)	1247 (35.87)	1596 (42.32)
<1 piece of fruit a day	1214 (5.85)	158 (3.55)	162 (5.54)	194 (4.92)	145 (6.64)	269 (7.74)	286 (7.58)
**Diabetes, n (%)**							
No	19,167 (89.014)	4378 (96.37)	2442 (80.51)	3623 (88.00)	2052 (88.30)	3224 (89.76)	3448 (88.59)
Yes	2334 (10.86)	165 (3.63)	591 (19.49)	494 (12.00)	272 (11.70)	368 (10.24)	444 (11.41)
**Desired emotional support, n (%)**							
Low	2241 (10.81)	228 (5.09)	326 (11.05)	321 (8.05)	185 (8.46)	712 (20.74)	469 (12.69)
Medium	7783 (37.54)	1415 (31.61)	1096 (37.15)	1427 (35.77)	953 (43.60)	1529 (44.54)	1363 (36.88)
High	10,706 (51.64)	2833 (63.29)	1528 (51.80)	2241 (56.18)	1048 (47.94)	1192 (34.72)	1864 (50.43)
**Stress at work last 12 months, n (.%)**							
Never	10,724 (50.72)	1694 (37.51)	1491 (49.75)	2176 (53.64)	1474 (65.34)	1769 (50.33)	2120 (55.76)
Sometimes	6990 (33.06)	2027 (44.88)	949 (31.66)	1310 (32.29)	589 (26.11)	1057 (30.07)	1058 (27.83)
Often	2315 (10.95)	667 (14.77)	328 (10.94)	385 (9.49)	128 (5.67)	426 (12.12)	381 (10.02)
Constantly	1114 (5.27)	128 (2.83)	229 (7.64)	186 (4.58)	65 (2.88)	263 (7.48)	243 (6.39)
**Stress at home last 12 months, n (%)**							
Never	10,724 (50.72)	1694 (37.51)	1491 (49.75)	2176 (53.64)	1474 (65.34)	1769 (50.33)	2120 (55.76)
Sometimes	6990 (33.06)	2027 (44.88)	949 (31.66)	1310 (32.29)	589 (26.11)	1057 (30.07)	1058 (27.83)
Often	2315 (10.95)	667 (14.77)	328 (10.94)	385 (9.49)	128 (5.67)	426 (12.12)	381 (10.02)
Constantly	1114 (5.27)	128 (2.83)	229 (7.64)	186 (4.58)	65 (2.88)	263 (7.48)	243 (6.39)
**Adverse life events last 12 months, n (%)**							
No	7269 (34.38)	1842 (40.79)	912 (30.43)	963 (23.74)	901 (39.94)	1263 (35.93)	1388 (36.51)
Yes	13,874 (65.62)	2674 (59.21)	2085 (69.57)	3094 (76.26)	1355 (60.06)	2252 (64.07)	2414 (63.49)

**Missing values** were <1 % for all variables except occupation 15 %.

**Occupational Level:** Occupational levels were determined using the International Standard Classification of Occupations (ISCO).

**Physical Activity:** Physical activity levels were assessed using the SQUASH questionnaire.

**Smoking:** Smoking status was determined through self-reported data.

**Alcohol Consumption:** Alcohol consumption was determined through self-reported data.

**Body Mass Index (BMI):** Body mass index was calculated from self-reported weight and height.

**Fruit Intake:** Fruit intake was determined through self-reported data.

**Diabetes:** Diabetes status was assessed based on WHO criteria, physician diagnosis, fasting blood glucose (FBG), and medication use.

**Stress at Work (Last 12 Months):** Stress at work during the past 12 months was assessed using the Interheart questionnaire.

**Stress at Home (Last 12 Months):** Stress at home during the past 12 months was assessed using the Interheart questionnaire.

**Adverse Life Events (Last 12 Months):** Adverse life events during the past 12 months were assessed using the Interheart questionnaire.

**Desired Social Support**: This was determined by combining the SSQT (Social Support Questionnaire Transactions) and SSQSa (Social Support Questionnaire Satisfaction) scores and categorizing them into low, medium, and high levels. These categories were derived using the methodology outlined by Muilwijk M, Bolijn R, Galenkamp H, Stronks K, van Charante EM, van Valkengoed IG in their study: The association between gender-related characteristics and type 2 diabetes risk in a multi-ethnic population: The HELIUS study. Nutrition, Metabolism and Cardiovascular Diseases. 2022 Jan 1;32(1):142–150.

### Proportions of stressors and type 2 diabetes

Stress at work (constant + several periods) was most reported among South-Asian Surinamese (25 %) and Turkish origin participants (19 %), while it was least common among Ghanaian (9 %) and Dutch origin individuals (13 %). Stress at home followed a similar pattern, with higher levels reported by South-Asian Surinamese (25 %) and Turkish participants (20 %), compared to 9 % of Ghanaians and 13 % of Dutch origin individuals**.** Exposure to an adverse life event in the past year was also elevated among several migrant groups, particularly African Surinamese (76 %) and Ghanaian origin participants (70 %), while Dutch origin individuals reported the lowest exposure (59 %). The most common adverse life event was housing problems, reported by 44 % of African Surinamese and 20 % of Dutch origin individuals. The least common was financial problems, reported by 5 % of Dutch and 14 % of Turkish origin individuals (Appendix 3).

In parallel, the prevalence of T2D was highest among South-Asian Surinamese (19 %) and African Surinamese (12 %), followed by Ghanaian (12 %), Moroccan (11 %), and Turkish origin individuals (12 %). Dutch origin participants had the lowest prevalence at 4 % (Table 1).

### Associations between stressors and type 2 diabetes

Our analyses were first conducted in the total population and then stratified by migration background, as interaction tests identified positive interactions between stress at work, stress at home, and migration background in their relationship with T2D (Appendix 4).

**Fig. 2:**
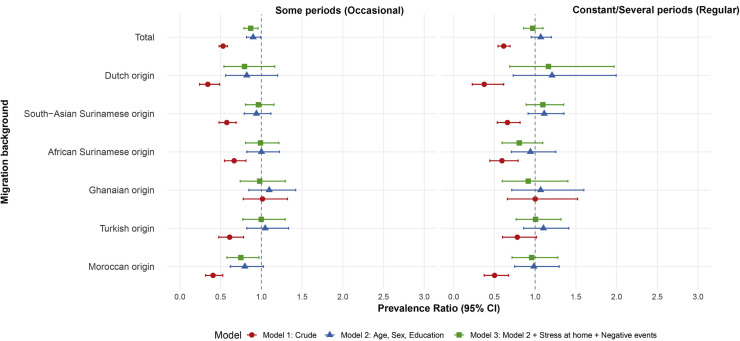
**Association between stress at work and type 2 diabetes.** This figure presents prevalence ratios (PRs) and 95 % confidence intervals from robust Poisson regression models assessing the association between stress at work and type 2 diabetes. Categories include "occasional" and "regular" stress at work, with "never" or no stress at work as the reference group.

In the total population, occasional and regular work-related stress (compared to none) showed a significant negative association with T2D in the crude models, but these associations disappeared in the regular group after adjusting for age, sex, education, stress at home, and adverse life events (Fig. 2). Across ethnic groups, the crude models revealed a significant negative association between work-related stress and T2D in most groups, except for Ghanaians. After adjustment, these associations disappeared in all groups except for the Moroccan origin participants (occasional work-related stress). Overall, occasional work-related stress was negatively associated with T2D prevalence in both the total population (adjusted Prevalence Ratio 0.82; 95 % CI 0.75–0.93) and the Moroccan origin group (aPR 0.76; 95 % CI 0.63–0.97) (Fig. 2).

**Fig. 3:**
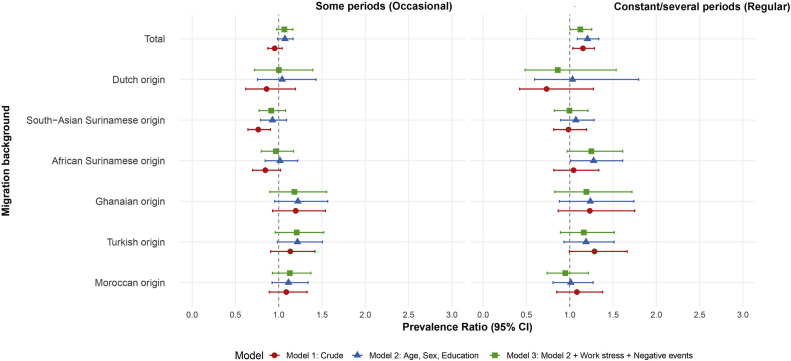
**Association between stress at home and type 2 diabetes.** This figure shows PRs and 95 % confidence intervals from robust Poisson regression models evaluating the association between stress at home and type 2 diabetes. The exposure categories are "occasional" and "regular" home stress, compared to "never" home stress as the reference.

In the total population, regular home stress (compared to none) showed a significant positive association with T2D in the crude models, but occasional home stress did not (Fig. 3). This positive association persisted after adjusting for age, sex, education, stress at work, and adverse life events. Across ethnic groups, the crude models revealed a significant negative association between occasional home stress and T2D in South-Asian Surinamese participants, but not in other groups, and regular home stress showed no significant association. After adjustment, these associations in South-Asian Surinamese participants disappeared. Overall, regular home stress was positively associated with T2D prevalence in the total population (adjusted PR 1.15; 95 % CI 1.03–1.28), but not across ethnic groups (Fig. 3).

**Fig. 4:**
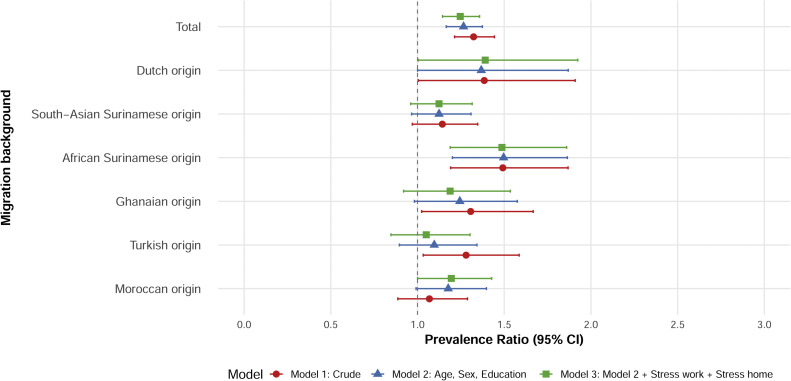
**Association between adverse life events and type 2 diabetes.** This figure displays PRs and 95 % confidence intervals from robust Poisson regression models estimating the association between experiencing at least one adverse life event in the previous 12 months and type 2 diabetes, with no reported events as the reference category.

Experiencing an adverse life event (compared to none) showed a positive association with T2D in both the crude and adjusted models in the total population (Fig. 4). The crude models revealed significant associations in Dutch, African Surinamese, Ghanaian, and Turkish origin participants. After adjustment, significant associations remained only in Dutch and African Surinamese participants. Overall, experiencing an adverse life event was positively associated with T2D in the total population (adjusted PR 1.22; 95 % CI 1.03–1.41), and in Dutch (adjusted PR 1.48; 95 % CI 1.21–1.76) and African Surinamese (adjusted PR 1.43; 95 % CI 1.09–1.89) origin groups (Fig. 4).

Model diagnostics for the fully adjusted Poisson regression showed no overdispersion (dispersion ratio ≤ 1), good fit (deviance residuals near zero), and no influential observations (Cook's distance) (Appendix 5).

### Mediation role of lifestyle factors

Mediation analyses examined the role of lifestyle factors in the relationship between psychosocial stressors and T2D in the total population and specific ethnic groups where associations between stressors and T2D were statistically significant in the original analysis (Appendix 6). BMI and alcohol consumption were the strongest mediators: alcohol use explained up to 17 % of the relationship for stress at work and up to 25 % for stress at home, while BMI explained 14 % for stress at work and 20 % for stress at home. The weakest mediation effects were observed for adverse life events, with each lifestyle factor mediating not >10 % of the relationship (Appendix 6).

### Moderation role of desired social support

In line with findings from Muilwijk et al.⁵ showing an inverse association between desired social support and T2D, we also observed that medium and high levels of desired support were associated with lower T2D prevalence prior to assessing interaction effects (Appendix 7). We then tested whether desired support moderated the relationship between psychosocial stressors and T2D in the total population and in ethnic groups with significant initial associations. No significant interactions were found (Appendix 8), and models for the Dutch group did not converge due to few participants reporting low support. However, when all stressors and desired support were included in a single model, previously observed associations—occasional home stress, regular work stress, and medium/high social support—were no longer statistically significant. Only adverse life events remained significantly associated with T2D in the total population (aPR 1.22; 95 % CI 1.06–1.40) and among African Surinamese participants (aPR 1.53; 95 % CI 1.11–2.15).

## Discussion

### Key findings

We examined the associations between three psychosocial stressors and T2D among migrant and non-migrant (Dutch) populations in The Netherlands. Occasional work-related stress was linked to a lower prevalence of T2D in the total population and among individuals of Moroccan origin. Regular home stress was associated with a higher T2D prevalence overall, with positive trend of effects in populations with migration background and inverse trend of effects in Dutch majority population. Adverse life events were associated with increased T2D risk in the total population, particularly among those of Dutch and African Surinamese origin. These associations were partially mediated by BMI and alcohol use. Desired social support buffered the impact of work and home stress on T2D, but not that of adverse life events.

### Discussion of key findings

We found that occasional work stress, compared to no work stress, was inversely associated with T2D in the total population and among individuals of Moroccan origin, while regular work stress showed no significant association. Previous studies have reported mixed results—some identifying a positive association between work stress and T2D (Nyberg et al., 2014), while others found no consistent link (Sui et al., 2016).

Mediation analyses revealed that BMI and alcohol use partially explained the association, but these mediators accounted for less than half of the total effect. This suggests that non-behavioral mechanisms may have a stronger protective influence. One possible explanation for these protective biological effects is the concept of hormesis (Calabrese and Baldwin, 2003), where exposure to low, non-chronic stress triggers adaptive physiological responses (Calabrese and Baldwin, 2003). It is possible that occasional mild stress, without prolonged activation of the hypothalamic–pituitary–adrenal (HPA) axis, may enhance resilience and support metabolic health (Calabrese and Baldwin, 2003). In contrast, regular work stress, compared to no work stress, showed no significant association with T2D, reinforcing the idea that only low-level stress may elicit such beneficial effects (Calabrese and Baldwin, 2003).

The lack of variation in the occasional work stress results across ethnic groups suggests that the inverse effect may be modest and primarily detectable in the pooled sample due to increased statistical power. Interestingly, the inverse association disappeared after adjusting for desired social support, indicating that strong emotional support may buffer stress so effectively that it prevents the physiological activation needed for beneficial adaptation. This may also explain why the Moroccan-origin group—who reported lower levels of desired social support—showed a significant association prior to adjustment, which attenuated after support was included in the model.

In contrast, regular home stress (compared to none) was positively associated with T2D in the total population, with positive trend of effects in populations with migration background and inverse trend of effects in Dutch majority population. On the other hand, occasional home stress showed no significant association with T2D. This finding aligns with previous research linking chronic home-based stress—such as caregiving demands, or family conflict—to prolonged HPA axis activation and metabolic disruption (Kelly and Ismail, 2015; Chilunga et al., 2023; Chilunga et al., 2019). The observed association likely reflects the cumulative physiological and behavioral effects of ongoing domestic stress, including elevated cortisol, unhealthy eating, and physical inactivity—key contributors to T2D risk (Kelly and Ismail, 2015). Mediation analyses supported these mechanisms. BMI accounted for approximately 20 % of the association between home stress and T2D, indicating that stress-related weight gain plays a key mediating role. However, as with work stress, mediation explained less than half of the total association, suggesting that other non-behavioral pathways may also be involved.

Unlike occasional work stress, occasional home stress did not exhibit a protective, hormetic effect. This may be due to differences in how stress is experienced and appraised in different contexts. Occasional work stress is often goal-oriented and may be followed by a sense of accomplishment or reward, fostering positive physiological adaptation (Hackett and Steptoe, 2017; Kelly and Ismail, 2015). In contrast, home stress is typically less goal oriented, making even low levels less likely to elicit adaptive benefits (no accomplishments gained afterwards) (Hackett and Steptoe, 2017; Kelly and Ismail, 2015).

As with work stress, the effect of home stress was modest and primarily detectable in the pooled population. However, the trend of stronger associations in migrant-origin populations suggests that regular home stress may have a more pronounced impact in these groups than in the Dutch majority. This difference, though subtle, may be partly explained by the higher levels of desired social support reported among the Dutch. Indeed, the association between regular home stress and T2D was attenuated after adjusting for social support, underscoring its buffering role in mitigating the physiological and behavioral consequences of chronic home-based stress.

Experiencing a negative life event was positively associated with T2D in the total population. These findings align with previous research linking significant life disruptions—such as illness, death of loved ones or legal problems—to overactivation of stress pathways, and subsequently T2D (Chilunga et al., 2023; Chilunga et al., 2019; Mooy et al., 2000). Mediation analyses showed that BMI and alcohol use accounted for a modest proportion of the association (9 % and 7 % respectively), suggesting that stress-related weight gain and alcohol consumption, plays a partial role.

The association of adverse life events with T2D was strongest among Dutch and African Surinamese origin participants, possibly due to their relatively high exposure to acute stressors compared to other ethnic groups. Among Dutch participants, the most frequently reported events were relationship issues (22 %) and housing problems (20 %), while in African Surinamese participants, these proportions were even higher, with 28 % reporting relationship issues and 44 % reporting housing problems. These stressors may be particularly impactful in these groups, contributing to overactivation of the hypothalamic–pituitary–adrenal (HPA) axis and, consequently, increased T2D risk (Chilunga et al., 2023; Chilunga et al., 2019; Mooy et al., 2000). Notably, adjustment for desired social support did not attenuate the association, suggesting that the effects of acute life events may be less amenable to emotional buffering than more chronic forms of stress and may exert a stronger influence on stress-related physiological pathways and T2D development.

This study carries significant implications for public health research, as it sheds light on the need to understand less commonly studied risk factors, such as psychosocial stress, particularly in-migrant groups. This endeavor could pave the way for innovative culturally adapted and tailored interventions targeting stress-related pathways to address the disproportionate T2D burden in migrant populations.

### Strengths and limitations

The main strength of this study is that it was performed in a multi-ethnic cohort, which enables comparison of psychosocial stress and T2D in migrant and non-migrant populations. In addition, the large sample size of this study made it possible to estimate robust effect sizes. However, this study also has limitations. The cross-sectional design does not allow for conclusions regarding the causal and temporal relationship between stress and T2D. Secondly, data was collected through self-reported questionnaires, which could lead to response bias in reporting stress. To mitigate recall bias, the questionnaires used a 12-month reference period, as studies suggest that memory recall tends to be more accurate over this timeframe. However, this approach does not capture the effects of stress experienced earlier in life or susceptibility to stress later in life. Thirdly, the duration of stress exposure was not measured, and we were unable to distinguish between acute and chronic stress. Fourth, work and home stress were each assessed using a single item from the INTERHEART scale (Appendix 1). While this instrument is validated and widely used, it may not fully capture the complexity of these stressors. However, it is widely used allowing for comparisons between studies. Lastly, stress may be perceived differently across ethnic groups, potentially leading to variations due to differences in understanding.

## Conclusion

Our study found that occasional home stress was associated with lower T2D risk among Moroccan origin participants, while regular work stress was not associated with higher T2D risk in any of the ethnic groups. Adverse life events showed positive associations with T2D specifically among Dutch and African Surinamese origin groups. These effects were partially mediated by BMI and alcohol use, indicating behavioral pathways. Desired social support buffered the effects of chronic stress at home and work but not those of acute life events, suggesting limited emotional protection against sudden stressors. These findings highlight the need for culturally relevant and tailored interventions to address chronic stress and strengthen social support to prevent T2D, especially in diverse populations.

## Funding

The HELIUS study is funded by the Dutch Heart Foundation (grant number 2010T084), Dutch Organization for Health Research and Development (grant number ZonMw 200500003), European Union (FP-7) (grant number 278901), and the European Fund for the Integration of non-EU immigrants (EIF) (grant number 2013EIF013).

## Availability of data and materials

The HELIUS data are owned by the Amsterdam University Medical Centers, location AMC in Amsterdam, The Netherlands. Any researcher can request the data by submitting a proposal to the HELIUS Executive Board as outlined at http://www.heliusstudy.nl/en/researchers/collaboration, by email: heliuscoordinator@amsterdamumc.nl. The HELIUS Executive Board will check proposals for compatibility with the general objectives, ethical approvals, and informed consent forms of the HELIUS study. There are no other restrictions to obtaining the data and all data requests will be processed in the same manner.

## Author contributions

All authors contributed substantially to this study and approved the submission. DDCG, CA and FPC contributed to the conception and design of the study. HG was involved in the acquisition and curation of the data. DDCG and FPC were responsible for analysing/interpreting statistical data and writing the text. Each author contributed important intellectual content, assisting in the interpretation of the results and providing critical reviews during the writing or revision of the article. FPC assumes responsibility that the study has been reported transparently and that no important aspects of the study have been omitted.

## CRediT authorship contribution statement

**Daniela Del Carlo Gonçalves:** Writing – original draft, Methodology, Formal analysis, Data curation, Conceptualization. **Charles Agyemang:** Conceptualization, Methodology, Validation, Writing – original draft, Writing – review & editing. **Eva L. van der Linden:** Writing – review & editing, Methodology, Investigation, Conceptualization. **Charles Hayfron Benjamin:** Writing – review & editing, Supervision. **Anja Lok:** Writing – review & editing, Validation, Supervision. **Henrike Galenkamp:** Writing – review & editing, Project administration, Methodology, Investigation. **Eric Moll van Charante:** Writing – review & editing, Supervision. **Felix P. Chilunga:** Writing – review & editing, Writing – original draft, Validation, Supervision, Methodology, Conceptualization.

## Declaration of competing interest

The authors declare that they have no known competing financial interests or personal relationships that could have appeared to influence the work reported in this paper.

## Appendix Supplementary materials

Image, application
1
