# Supplementary material for: Impact of psychosocial stressors on type 2 diabetes among migrants and non-migrants in The Netherlands: The HELIUS study
Source: J Migr Health. 2025 Mar 31;11:100330. doi: 10.1016/j.jmh.2025.100330 (PMC11999673; doi:10.1016/j.jmh.2025.100330)
Supplement: Supplementary file 1 [file mmc1.pdf]

**Appendix 1- Psychological scale**

| | |
|---|---|
| In the past 12 months, have you felt stressed (feeling irritable or anxious or having trouble sleeping) because of the situation at work? | □ Never<br>□ Some periods<br>□ Several periods<br>□ Constantly<br>□ Doesn't apply |
| In the past 12 months, have you felt stressed (feeling irritable or anxious or having trouble sleeping) because of the situation at home? | □ Never<br>□ Some periods<br>□ Several periods<br>□ Constantly |

**Appendix 2- List of threatening experiences (LTE)**

| We will now mention some events. Please indicate whether you've experienced these events in the past 12 months. | No | Yes |
|---|---|---|
| a. You suffered from a serious illness or injury. | □ | □ |
| b. A close relative had a serious illness or injury. | □ | □ |
| c. Your parent, child, brother, sister, or spouse died. | □ | □ |
| d. Another relative (such as an aunt, cousin, or grandparent) or close friend died. | □ | □ |
| e. You broke off a steady relationship. | □ | □ |
| f. A long-term friendship with a good friend or family member was broken off. | □ | □ |
| g. You had a serious problem with a good friend, family member, or neighbour. | □ | □ |
| h. You were sacked from your job or became unemployed. | □ | □ |
| i. You had a major financial crisis | □ | □ |

**Appendix 3.** Types of adverse life events in the last 12 months.

| Life Event | Dutch origin | South-Asian Surinamese origin | African Surinamese origin | Ghanaian origin | Turkish origin | Moroccan origin |
|---|---|---|---|---|---|---|
| **Financial problems** | | | | | | |
| Yes | 237 (5.27%) | 387 (12.98%) | 424 (10.54%) | 262 (11.74%) | 494 (14.14%) | 482 (12.75%) |
| No | 4257 (94.73%) | 2595 (87.02%) | 3600 (89.46%) | 1970 (88.26%) | 3000 (85.86%) | 3299 (87.25%) |
| **Relationship issues** | | | | | | |
| Yes | 985 (21.91%) | 842 (28.24%) | 1141 (28.34%) | 296 (13.28%) | 769 (22.05%) | 932 (24.69%) |
| No | 3510 (78.09%) | 2140 (71.76%) | 2885 (71.66%) | 1933 (86.72%) | 2718 (77.95%) | 2843 (75.31%) |
| **Work-related problems** | | | | | | |
| Yes | 247 (5.5%) | 295 (9.89%) | 492 (12.27%) | 387 (17.36%) | 252 (7.21%) | 312 (8.27%) |
| No | 4246 (94.5%) | 2687 (90.11%) | 3519 (87.73%) | 1842 (82.64%) | 3241 (92.79%) | 3462 (91.73%) |
| **Housing problems** | | | | | | |
| Yes | 893 (19.88%) | 1016 (34.08%) | 1759 (43.75%) | 656 (29.5%) | 831 (23.82%) | 1008 (26.69%) |
| No | 3600 (80.12%) | 1965 (65.92%) | 2262 (56.25%) | 1568 (70.5%) | 2658 (76.18%) | 2768 (73.31%) |
| **Health problems** | | | | | | |
| Yes | 258 (5.74%) | 253 (8.52%) | 449 (11.23%) | 204 (9.18%) | 234 (6.71%) | 223 (5.91%) |
| No | 4233 (94.26%) | 2718 (91.48%) | 3549 (88.77%) | 2018 (90.82%) | 3252 (93.29%) | 3549 (94.09%) |
| **Family illness** | | | | | | |
| Yes | 353 (7.86%) | 491 (16.48%) | 743 (18.49%) | 284 (12.75%) | 501 (14.34%) | 497 (13.16%) |
| No | 4138 (92.14%) | 2489 (83.52%) | 3275 (81.51%) | 1944 (87.25%) | 2992 (85.66%) | 3280 (86.84%) |

| Life Event | Dutch origin | South-Asian Surinamese origin | African Surinamese origin | Ghanaian origin | Turkish origin | Moroccan origin |
|---|---|---|---|---|---|---|
| **Bereavement** | | | | | | |
| Yes | 359 (7.99%) | 361 (12.11%) | 502 (12.49%) | 246 (11.05%) | 369 (10.57%) | 368 (9.74%) |
| No | 4132 (92.01%) | 2621 (87.89%) | 3517 (87.51%) | 1981 (88.95%) | 3122 (89.43%) | 3409 (90.26%) |
| **Violence or crime** | | | | | | |
| Yes | 404 (9%) | 342 (11.49%) | 548 (13.65%) | 270 (12.12%) | 412 (11.83%) | 430 (11.39%) |
| No | 4086 (91%) | 2635 (88.51%) | 3468 (86.35%) | 1958 (87.88%) | 3070 (88.17%) | 3345 (88.61%) |
| **Legal problems** | | | | | | |
| Yes | 324 (7.21%) | 561 (18.83%) | 977 (24.35%) | 558 (25.09%) | 938 (26.88%) | 682 (18.1%) |
| No | 4168 (92.79%) | 2418 (81.17%) | 3036 (75.65%) | 1666 (74.91%) | 2551 (73.12%) | 3086 (81.9%) |

**Appendix 4.** Interactions between migration status and stress factors in the relationship with type 2 diabetes

| **Stress Level** | **Dutch Origin** | **South-Asian Surinamese Origin** | **African Surinamese Origin** | **Ghanaian Origin** | **Turkish Origin** | **Moroccan Origin** |
|---|---|---|---|---|---|---|
| **Stress at work (last 12 months)** | | | | | | |
| Never | Ref | Ref | Ref | Ref | Ref | Ref |
| Seldom | Ref | **1.47 (1.01-2.16)** | **1.55 (1.05-2.32)** | **1.65 (1.07-2.55)** | 1.44 (0.95-2.19) | 1.11 (0.73-1.69) |
| Regularly/Often | Ref | 1.29 (0.77-2.17) | 1.07 (0.62-1.87) | 1.15 (0.61-2.15) | 1.15 (0.67-1.98) | 1.01 (0.58-1.75) |
| **Stress at home (last 12 months)** | | | | | | |
| Never | Ref | Ref | Ref | Ref | Ref | Ref |
| Sometimes | Ref | 0.98 (0.69-1.39) | 1.09 (0.76-1.58) | 1.28 (0.86-1.92) | 1.25 (0.85-1.82) | 1.17 (0.81-1.69) |
| Regularly/Often | Ref | 1.18 (0.66-2.10) | 1.48 (0.82-2.68) | **1.35 (1.11-2.57)** | **1.32 (1.02-2.39)** | 1.15 (0.63-2.07) |
| **Negative life events** | | | | | | |
| No | Ref | Ref | Ref | Ref | Ref | Ref |
| Yes | Ref | 0.85 (0.62-1.20) | 1.13 (0.77-1.66) | 0.95 (0.64-1.41) | 0.84 (0.58-1.21) | 0.88 (0.61-1.25) |

**Ref**= reference category (Never/no for stressors and Dutch origin for ethnic groups)

## Appendix 5. Model fit assessment (fully adjusted model)

| Migration Background | Pearson Chi-square | Residual DF | Dispersion Ratio | Mean Deviance Residual | Min Deviance Residual | Median Deviance Residual | Max Deviance Residual | Influential Observations (cooks' distance) |
|---|---|---|---|---|---|---|---|---|
| Total Population | 16113.22 | 21012.0 | 0.7669 | -0.199 | -1.2923 | -0.3141 | 2.7614 | 0 |
| Dutch origin | 3461.436 | 4483.0 | 0.7721 | -0.1351 | -1.0042 | -0.1521 | 3.223 | 0 |
| African Surinamese origin | 2166.174 | 2976.0 | 0.7279 | -0.2134 | -1.3625 | -0.3871 | 2.4319 | 0 |
| South-Asian Surinamese origin | 3344.205 | 4018.0 | 0.8323 | -0.21 | -1.1715 | -0.3712 | 2.7709 | 0 |
| Ghanaian origin | 1826.091 | 2223.0 | 0.8215 | -0.2182 | -1.0047 | -0.3966 | 2.6609 | 0 |
| Moroccan origin | 2663.018 | 3486.0 | 0.7639 | -0.1925 | -1.4162 | -0.2913 | 2.6408 | 0 |
| Turkish origin | 2500.753 | 3771.0 | 0.6632 | -0.1881 | -1.4204 | -0.2596 | 2.507 | 0 |

Assessment of model fit of stress factors vs type 2 diabetes adjusted for age, sex and education. Model is robust position regression

**Pearson Chi-square**: Measures goodness of fit; higher values indicate greater deviation from expected values.

**Residual DF**: Degrees of freedom left after fitting the model.

**Dispersion Ratio:** Ratio of Pearson Chi-square to DF; values near 1 indicate appropriate model fit.

**Mean Deviance Residual:** Average deviation from the predicted values; closer to zero is ideal.

**Min Deviance Residual:** Lowest observed residual value, indicating extreme underprediction.

**Median Deviance Residual:** The middle value of residuals, representing central tendency.

**Max Deviance Residual**: Highest observed residual value, indicating extreme overprediction.

**Cook's Distance:** Identifies influential observations; higher values indicate greater influence on model estimates. Observations exceeding the threshold (4/n, where n = total observations per subgroup) are considered influential and potential outliers requiring further inspection.

**Appendix 6:** Mediation role of lifestyle factors in the relationship between stress factors and type 2 diabetes

| Stressor | Mediator | Direct Effect (PR) | Indirect Effect (PR) | Total Effect (PR) | Proportion Mediated (%) |
|---|---|---|---|---|---|
| **Occasional stress at work (Total population)** | | | | | |
| | Fruit Intake | 0.999 | 0.999 | 0.999 | 6.9% |
| | Physical Activity | 0.999 | 1.000 | 0.999 | 6.3% |
| | Smoking | 1.000 | 0.999 | 0.999 | -0.1% |
| | Alcohol Use | 0.996 | 1.002 | 0.998 | 16.6% |
| | BMI | 0.998 | 1.001 | 1.000 | 14.3% |
| **Occasional stress at work (Moroccan origin)** | | | | | |
| | Fruit Intake | 0.984 | 1.000 | 0.983 | 0.8% |
| | Physical Activity | 0.985 | 1.000 | 0.984 | 3.0% |
| | Smoking | 0.982 | 1.000 | 0.982 | 2.6% |
| | Alcohol Use | 0.983 | 0.993 | 0.978 | 11.6% |
| | BMI | 0.983 | 0.999 | 0.982 | 5.2% |
| **Regular stress at home (Total population)** | | | | | |
| | Fruit Intake | 0.999 | 1.008 | 1.007 | -9.1% |
| | Physical Activity | 1.000 | 1.009 | 1.008 | -3.5% |
| | Smoking | 1.000 | 1.007 | 1.007 | -1.4% |
| | Alcohol Use | 0.997 | 1.010 | 1.007 | -24.8% |
| | BMI | 1.002 | 1.006 | 1.008 | 19.7% |
| **Adverse life events (Total population)** | | | | | |
| | Fruit Intake | 1.000 | 1.025 | 1.025 | 0.5% |
| | Physical Activity | 1.000 | 1.026 | 1.026 | -1.4% |
| | Smoking | 1.000 | 1.025 | 1.025 | -1.3% |
| | Alcohol Use | 0.998 | 1.031 | 1.028 | -7.2% |
| | BMI | 1.002 | 1.022 | 1.025 | 8.9% |
| **Adverse life events (Dutch origin)** | | | | | |
| | Fruit Intake | 1.010 | 1.000 | 1.010 | 0.0% |
| | Physical Activity | 1.013 | 1.000 | 1.012 | -2.8% |
| | Smoking | 1.011 | 1.000 | 1.011 | -1.1% |
| | Alcohol Use | 1.010 | 1.000 | 1.010 | 0.2% |
| | BMI | 1.009 | 1.001 | 1.010 | 7.8% |
| **Adverse life events (African Surinamese origin)** | | | | | |
| | Fruit Intake | 1.043 | 1.000 | 1.043 | -0.8% |
| | Physical Activity | 1.044 | 1.000 | 1.044 | -0.4% |
| | Smoking | 1.044 | 0.999 | 1.042 | -2.2% |
| | Alcohol Use | 1.043 | 1.000 | 1.042 | -1.2% |
| | BMI | 1.042 | 1.003 | 1.045 | 5.5% |

Mediation role of lifestyle factors in the relationship between psychosocial stressors and type 2 diabetes (T2D). Presented are prevalence ratios (PR) for direct, indirect (mediated via lifestyle factors), and total effects, along with the proportion mediated (%). Results are shown for the total population and specifically for ethnic groups where associations between stressors and T2D were statistically significant in the original analysis. Positive percentages indicate that lifestyle factors partially explain (mediate) the relationship between stressors and T2D, whereas negative percentages represent inverse mediation effects.

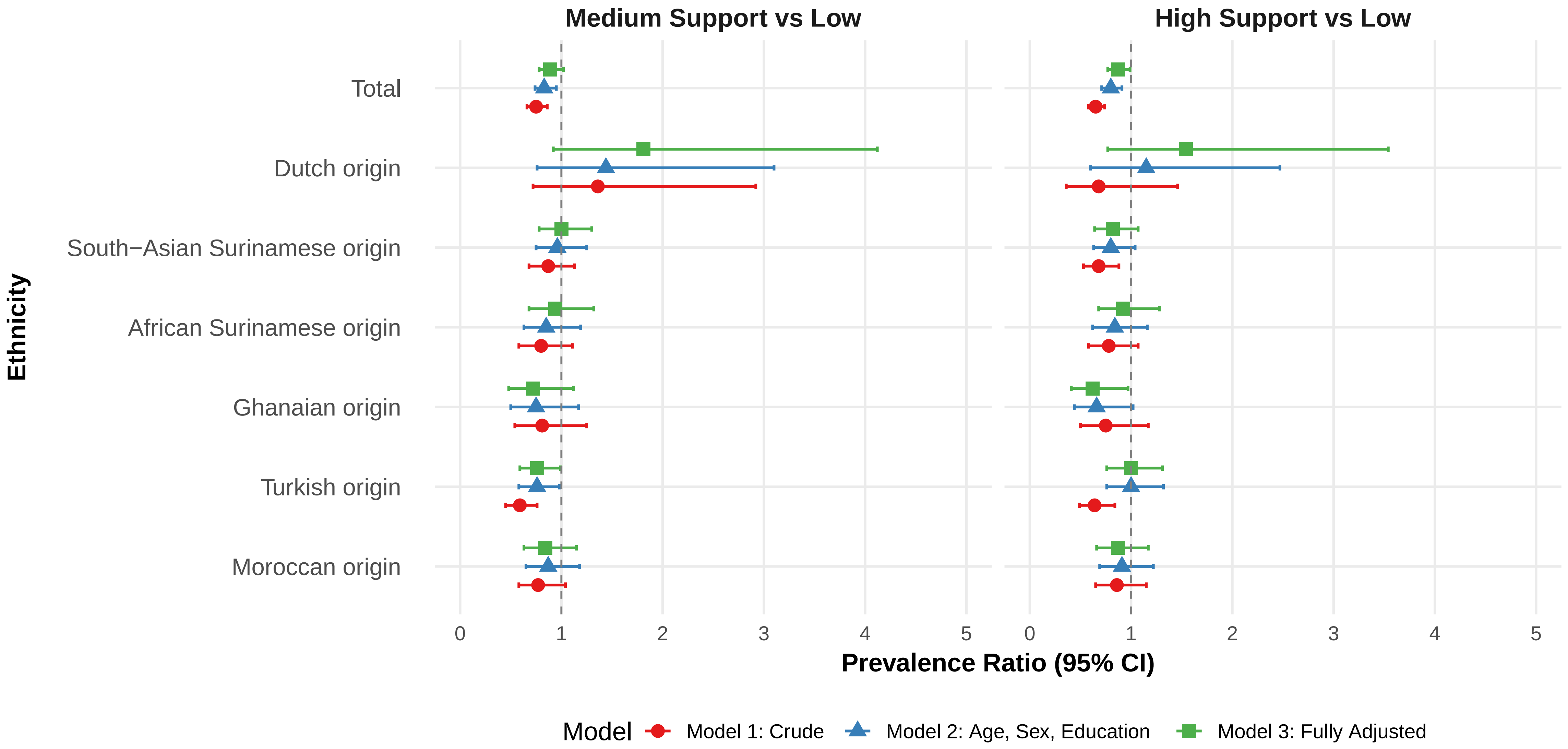
Medium Support vs Low
High Support vs Low
Total
Dutch origin
South−Asian Surinamese origin
African Surinamese origin
Ghanaian origin
Turkish origin
Moroccan origin
Ethnicity
0
1
2
3
4
5
Prevalence Ratio (95% CI)
Model
Model 1: Crude
Model 2: Age, Sex, Education
Model 3: Fully Adjusted

**Appendix 8:** Moderating Role of Social Support in the Relationship Between Stress Factors and Type 2 Diabetes

| Variable/population | Effect type | Prevalence Ratios | P-values |
|---|---|---|---|
| **Total population** | | | |
| Occasional work stress | Main | 0.79 (0.67-1.94) | 0.303 |
| Regular home stress | Main | 0.95 (0.76-1.18) | 0.580 |
| Adverse life events | Main | **1.22 (1.06-1.40)** | **0.002** |
| Medium social support | Main | 0.90 (0.62-1.26) | 0.512 |
| High social support | Main | 0.94 (0.79-1.11) | 0.421 |
| Occasional work stress × Medium support | Interaction | 1.27 (0.88-1.80) | 0.149 |
| Occasional work stress × High support | Interaction | 1.12 (0.90-1.41) | 0.264 |
| Regular home stress × Medium support | Interaction | 1.28 (0.86-1.90) | 0.152 |
| Regular home stress × High support | Interaction | 1.24 (0.93-1.66) | 0.093 |
| Adverse life events × Medium support | Interaction | 1.26 (0.88-1.84) | 0.164 |
| Adverse life events × High support | Interaction | 1.01 (0.83-1.23) | 0.923 |
| **Moroccan origin** | | | |
| Occasional work stress | Main | 0.85 (0.56-1.25) | 0.379 |
| Medium social support | Main | 0.99 (0.64-1.47) | 0.943 |
| High social support | Main | 0.97 (0.77-1.23) | 0.792 |
| Occasional work stress × Medium support | Interaction | 1.27 (0.55-2.79) | 0.498 |
| Occasional work stress × High support | Interaction | 0.67 (0.37-1.20) | 0.142 |
| **African Surinamese origin** | | | |
| Adverse life events | Main | **1.53 (1.11-2.15)** | **0.007** |
| Medium social support | Main | 1.26 (0.38-3.13) | 0.634 |
| High social support | Main | 1.09 (0.70-1.70) | 0.675 |
| Adverse life events × Medium support | Interaction | 0.98 (0.37-3.44) | 0.977 |
| Adverse life events × High support | Interaction | 0.94 (0.57-1.53) | 0.770 |

Associations of stress factors, desired social support, and their interaction (i.e., moderation effects) with type 2 diabetes (T2D). Presented are prevalence ratios (PRs) with 95% confidence intervals (CI) and p-values for both main effects and interaction terms. Results are shown for the total population and for ethnic groups where associations between stressors and T2D were statistically significant in the original analysis. Among Dutch participants, assessment of interaction (moderation) effects was not feasible due to the low number of individuals reporting low levels of social support. Interaction terms evaluate whether the relationship between psychosocial stressors and T2D differs depending on the level of desired social support.
